# Economic burden of comorbidities in psoriasis patients in the United States: results from a retrospective U.S. database

**DOI:** 10.1186/s12913-017-2278-0

**Published:** 2017-05-08

**Authors:** Steven R. Feldman, Haijun Tian, Isabelle Gilloteau, Patrick Mollon, Meng Shu

**Affiliations:** 1 Department of Dermatology, Wake Forest University School of Medicine, 4618 Country Club Road, Winston-Salem, North Carolina USA; 2 Novartis Pharmaceuticals Corporation, East Hanover, NJ USA; 3 Novartis Pharma AG, Basel, Switzerland; 4 Beijing Novartis Pharma Co. Ltd., Beijing, China

**Keywords:** Psoriasis, Costs, Comorbidities

## Abstract

**Background:**

Psoriasis is a multifactorial, inflammatory, skin disease associated with various comorbidities. The cost of those comorbidities is not well characterized. The present study assesses the incremental burden of comorbidities on healthcare resource utilization, direct costs and indirect costs associated with short-term disabilities among patients with psoriasis in the United States.

**Methods:**

A retrospective, U.S. cohort analysis was conducted using a large claims database. Adult psoriasis patients with at least two diagnoses of psoriasis during the years 2010 and 2011 (one psoriasis diagnosis had to happen in the year 2010) and with continuous enrollment of medical and pharmacy benefits in the years 2010 and 2011 were included. Psoriasis patients were categorized and compared according to the presence or absence of pre-selected comorbidities in the year 2010. Adjusted annual direct (costs associated with outpatient, emergency room, and inpatient claims, and outpatient pharmacy claims) and indirect costs (short-term disabilities) was assessed in patients with and without comorbidities using a regression analysis, controlling for age, gender, and psoriasis severity in year 2010.

**Results:**

In total, 56,406 patients (mean [SD]) age, 51.6 [14.6] years) were included in the analysis. The most prevalent comorbidities were hypertension (34.3%), hyperlipidemia (33.5%), cardiovascular disease (17.7%), diabetes (14.2%), and psoriatic arthritis (9.9%). Psoriasis patients with comorbidities used more healthcare resources than those without comorbidities. The incidence rate ratio (IRR) (95% CI) for patients with cardiovascular disease was 1.5 (1.4 − 1.5) for outpatient visits, 2.6 (2.4 − 2.8) for hospitalizations, and 2.3 (2.2 − 2.5) for ER visits, showing higher IRRs across all three types of resource use. The mean annual adjusted direct cost differences (i.e., incremental adjusted costs) in psoriasis patients with and without comorbidities were $9914.3, $8386.5, and $8275.1 for psoriatic arthritis, peripheral vascular disease, and cardiovascular disease, respectively. The mean annual incremental adjusted indirect costs of short-term disabilities were $1333, $1195, $994.9, and $996.6 for cerebrovascular disease, obesity, peripheral vascular disease, and depression, respectively.

**Conclusion:**

The presence of comorbidities was associated with higher healthcare resource utilization and costs among patients with psoriasis.

## Background

Psoriasis is a chronic, immune-mediated disease characterized by inflamed skin lesions accompanied by pain and itching [13]. In the United States (US), psoriasis affects more than 3.2% of the population or 7.4 million adults [31], with the onset most frequently occurring between 15 and 35 years of age [25, 27, 29]. Psoriasis places a substantial economic burden on both patient and society. A recent study reported the annual direct and indirect costs of psoriasis at approximately $112 billion in 2013, which included up to $63.2 billion of direct costs (expenses from the primary disease) and $35.4 billion of indirect costs (determined by loss of work productivity). The cost of medical conditions associated with psoriasis was estimated at $36.4 billion, and patients with psoriasis would pay a lifetime cost of $11,498 for relieving physical symptoms and emotional health; however, there was limited literature around intangible cost data (willingness to pay for global well-being and alleviate sufferings from the disease) [6].

Psoriasis is also associated with multiple comorbidities including psoriatic arthritis (PsA) [15], obesity [1], hypertension [2, 15], malignancies [15], metabolic syndrome [3, 5, 26], and cardiovascular diseases [2, 19, 28, 34]. Patients with psoriasis have an increased risk of depression/anxiety [7, 9, 23, 32] and suicidality [16, 25]. Although psoriasis is physically and psychologically devastating on its own, the presence of comorbidities increases the burden of the disease [4, 21, 22, 24, 30, 39]. Moreover, the burden of comorbidities increases with increasing psoriasis severity [20, 39]. Psoriasis patients with comorbidities are more likely to require urgent care, have greater hospitalization rates and more frequent outpatient visits, and incur greater costs than psoriasis patients without comorbidities [21]. Comorbidities result in higher overall pharmacy, and medical costs (adjusted annual cost differences per patient $17,696, and $5077, respectively) [12]. In addition, in a recent study conducted in the US in psoriasis and PsA patients, 92% of unemployed participants reported that they could not work solely due to psoriasis or PsA, revealing considerable work productivity burden of these diseases [4].

Studies assessing the economic burden associated with comorbidities in psoriasis patients remain limited. The objective of this study was to assess the incremental burden of comorbidities on healthcare resource utilization, direct costs and indirect costs associated with short-term disabilities among patients with psoriasis in the ﻿US.

## Methods

### Data source and sample selection

This retrospective cohort study was based on real-world data from the MarketScan® Commercial and Medicare Supplemental and Coordination of Benefits databases between January 01, 2010, and December 31, 2011 and from the MarketScan Health and Productivity Management (HPM) database between January 1, 2011, and December 31, 2011.

The Commercial and Medicare supplemental Databases includes healthcare expenditures of enrolled individuals in commercial health insurance plans sponsored by more than 100 large- and medium-sized employers in the US. It informs on patients’ characteristics including their comorbidity profile as well information regarding monthly enrollment data, hospitalization and outpatient medical claims, and outpatient prescription drug claims. The database is fully linkable to employees’ corresponding medical and pharmacy claims data.

The MarketScan HPM Database contains workplace absenteeism, short-term disabilities, and workers’ compensation data for a subset of enrollees in the Commercial Database. For short-term disabilities, data collected consist of primary reason for short-term disabilities (e.g., psoriasis), the date the episode of disability started, the date the employee returned to work (if applicable), and the payment to the disabled employee.

**Fig. 1:**
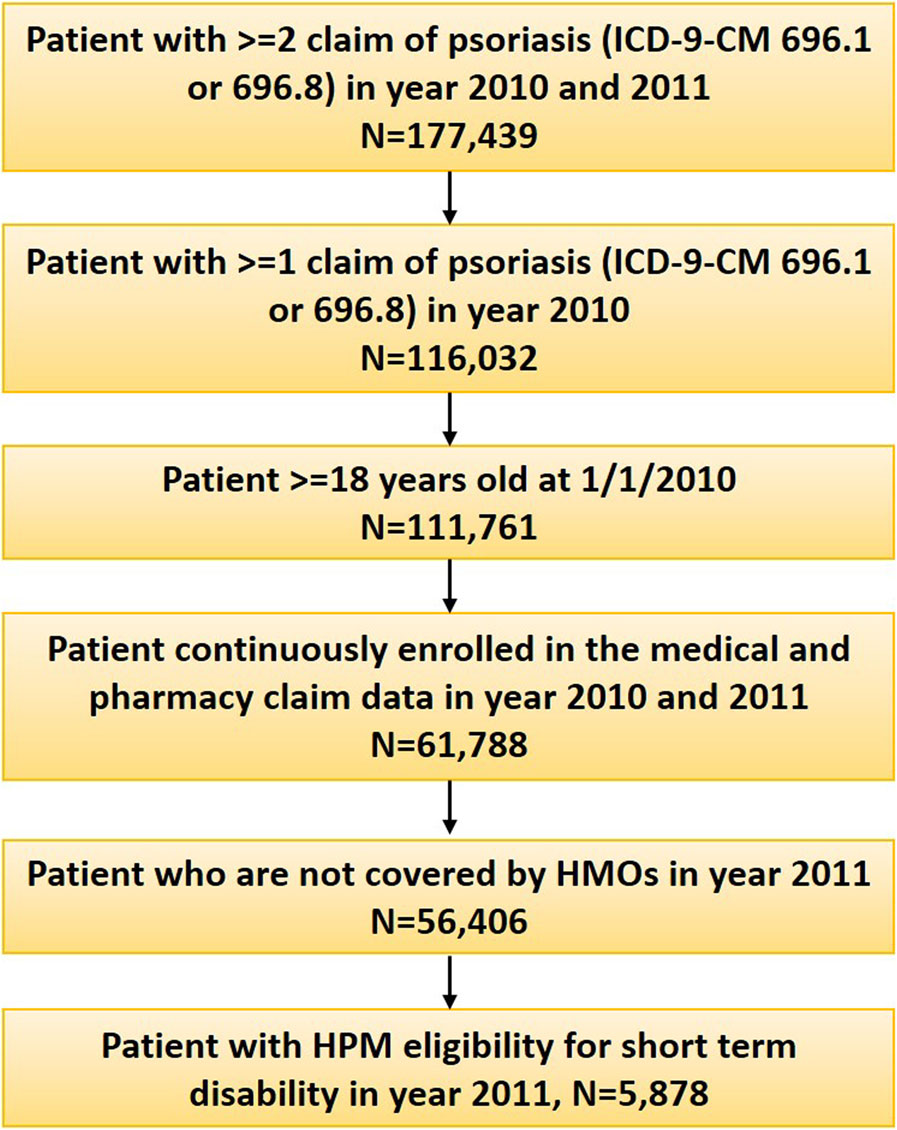
Patient selection scheme. HMO: Health Maintenance Organization; HPM: Health and Productivity Management; ICD: International Classification of Diseases

The study included adult patients (aged ≥18 years) with at least two diagnoses of Psoriasis (International Classification of Diseases, 9th Revision, Clinical Modification [ICD-9-CM]: 696.1, 696.8) on different dates between January 01, 2010, and December 31, 2011, with at least one psoriasis diagnosis in the year 2010 to ensure patients has psoriasis in year 2010. All patients are required to have continuous enrollment of medical and pharmacy benefits in the years 2010 and 2011. As HPM data was provided at annual bases, to maximize the sample size, we used calendar year to design the study. Year 2010 data was used to define psoriasis cohort, comorbidities and baseline characteristics, and year 2011 data was used to measure healthcare utilization and costs, and indirect costs associated with short-term disabilities longitudinally. Patients covered by Health Maintenance Organization (HMO) in year 2011 were excluded from healthcare cost analysis. As for indirect costs short- term disability analysis, only subset of patients with HPM eligibility for short- term disabilities in year 2011 were included in the analysis (Fig. 1).

### Variables

Age, gender, region, Charlson Comorbidity Index (CCI), and psoriasis disease severity were measured in the baseline year (i.e., year 2010). Psoriasis severity was defined according to patients’ treatment type in the baseline period; patients who received at least one systemic therapy or phototherapy in the baseline period comprised patients with moderate to severe psoriasis. All other psoriasis patients were defined as mild. The comorbidities of interest selected in this analysis were based on existing published research and consisted of PsA, cardiovascular disease, depression, anxiety, diabetes, hyperlipidemia, hypertension, obesity, cerebrovascular disease, and peripheral vascular disease [3, 11, 14, 15, 17, 21].

The direct costs were derived from the (not cause related) costs associated with outpatient, emergency room, and inpatient claims, and outpatient pharmacy claims as assessed in the year 2011. Indirect costs associated with short-term disabilities were assessed in the year 2011 using the Human Capital Approach [37]. These were estimated by multiplying number of short- term disability days, 8 h per day, and an average hourly wage, i.e. an average hourly wage data for all private industries from the US Bureau of Labor Statistics for the year 2011 [10].

### Statistical analysis

Descriptive statistics were used for all the aforementioned variables. For continuous variables, means and standard deviations were reported; for categorical variables, counts and percentages were reported. A bivariate analysis was conducted to describe the proportion of the pre-selected comorbidities and patient characteristics by psoriasis severity in the year 2010 (moderate to severe vs. mild).

Multiple regression were performed to compare utilization and adjusted direct/indirect costs in the year 2011 between the patients with and without comorbidities, controlling for age, gender, region, and psoriasis severity in the year 2010. For health care utilization, Poisson regression models were used, and the adjusted incidence rate ratios (IRRs) with respective 95% confidence intervals (CIs) and *p* values were reported for patients with and without comorbidities. For direct and indirect costs associated with short- term disabilities, two-part models (first with logistic regression for patients with positive indirect costs and second with gamma regression to model indirect positive costs) were used.

To estimate the incremental cost to comorbidities, the adjusted cost difference (change in the response by a change in a covariate) between patients with and without comorbidity was calculated using the recycled prediction method [38]. The adjusted costs for patients with each comorbidity were predicted based on the estimated two-part model by assuming all patients had such comorbidity (regardless of whether they had the comorbidity or not) while keeping other covariates constant. The adjusted costs for patients without the comorbidity were predicted using similar methodology assuming all patients had no such comorbidity. Thus, we compared two hypothetical populations (one with a comorbidity and the other without) that had the exact same values for patient characteristics in the model. The difference in adjusted costs between patients with comorbidities compared to those without comorbidities represented the incremental economic burden. The 95% CIs were calculated using bootstrapping method for both direct and indirect costs. The analysis was performed in SAS 9.3 (SAS Institute Inc., Cary, NC).

## Results

After applying the inclusion criteria, a total of 56,406 patients were included in the direct cost analysis, and 5878 in the indirect cost analysis (Fig. 1). The mean (standard deviation [SD]) age of the patients was 51.6 (14.6) years, 50% were male, and the mean CCI was 0.4 (0.9) (Table 1). Most patients (75.7%) had mild psoriasis, whereas 24.3% had moderate to severe psoriasis. Hypertension (34.3%), hyperlipidemia (33.5%), cardiovascular disease (17.7%), diabetes (14.2%), and PsA (9.9%) were the most prevalent comorbidities in the psoriasis population taken from this dataset. Patients with moderate to severe psoriasis had higher occurrence of at least one comorbidity than those with mild psoriasis (*p* < 0.0001). Comorbidities including PsA, depression, diabetes and obesity were more frequent in moderate to severe psoriasis vs. mild patients (21.2 vs. 6.2; *p* < 0.0001; (8.5 vs. 7.6; *p* = 0.0005), (15.9 vs. 13.7; *p* < 0.0001), and (5.2 vs. 4.8; *p* = 0.0687), respectively (Table 1).

**Table 1:** Patient demographics and comorbidities by severity of psoriasis

Baseline characteristics in year 2010	Total	Moderate to severe	Mild	*p* value
	(*N* = 56,406; 100%)	(*N* = 13,698; 24.3%)	(*N* = 42,708; 75.7%)	
Age (years), mean (SD)	51.63 (14.59)	51.79 (13.95)	51.58 (14.79)	<0.0001
Male, n (%)	28,208 (50.01)	6717 (49.04)	21,491 (50.32)	0.0089
Female (%)	28,198 (49.99)	6981 (50.96)	21,217 (49.68)	
Region				
North East	10,485 (18.59)	2478 (18.09)	8007 (18.75)	0.3327
North Central	16,528 (29.30)	3988 (29.11)	12,540 (29.36)	
South	20,633 (36.58)	5076 (37.06)	15,557 (36.43)	
West	8660 (15.35)	2129 (15.54)	6531 (15.29)	
Missing	100 (0.18)	27 (0.20)	73 (0.17)	
Charlson Comorbidity Index, mean (SD)	0.38 (0.93)			
Comorbidities, n (%)	Total	Moderate to severe	Mild	*p* value
	(*n* = 56,406)	(*n* = 13,698; 24.3%)	(*n* = 42,708; 75.7%)	
Psoriatic arthritis	5557 (9.85)	2901 (21.18)	2656 (6.22)	<0.0001
Cardiovascular disease	9992 (17.71)	2390 (17.45)	7602 (17.80)	0.3476
Depression	4388 (7.78)	1161 (8.48)	3227 (7.56)	0.0005
Anxiety	3148 (5.58)	734 (5.36)	2414 (5.65)	0.1923
Diabetes	8031 (14.24)	2181 (15.92)	5850 (13.70)	<0.0001
Hyperlipidemia	18,911 (33.53)	4572 (33.38)	14,339 (33.57)	0.6703
Hypertension	19,365 (34.33)	4775 (34.86)	14,590 (34.16)	0.1350
Obesity	2755 (4.88)	709 (5.18)	2046 (4.79)	0.0687
Cerebrovascular disease	2122 (3.76)	462 (3.37)	1660 (3.89)	0.0059
Peripheral vascular disease	2032 (3.60)	457 (3.34)	1575 (3.69)	0.0547
Any of the above comorbidities^a^	35,644 (63.19)	9294 (67.85)	26,350 (61.70)	<0.0001

^a^Any of the above comorbidities means if patients have any of the either comorbidities mentioned above. The percentages do not add up as they are not mutually exclusive

### Healthcare resource utilization

Psoriasis patients with comorbidities used more healthcare resources annually compared with those without comorbidities (IRR greater than one with *p* < 0.001 for almost all comorbidities). The highest IRR (95% CI) was observed for cardiovascular disease for outpatient visits (1.5 [1.4 − 1.5]), hospitalizations (2.6 [2.4 − 2.8]), and ER visits (2.3 [2.2 − 2.5]). Psoriasis patients with any comorbidity were more likely to use healthcare resources (Table 2).

**Table 2:** Adjusted incidence rate ratio for healthcare utilization in cohort of psoriasis patients with comorbidities

Comorbidity	Outpatient visits IRR Estimate	Lower CL	Upper CL	*p* value	Hospitalisation visits IRR Estimate	Lower CL	Upper CL	*p* value	Emergency Room Visits IRR Estimate	Lower CL	Upper CL	*P* value
Psoriatic arthritis	1.30	1.27	1.32	<0.0001	1.40	1.27	1.55	<0.0001	1.36	1.26	1.46	<0.0001
Cardiovascular disease	1.51	1.48	1.53	<0.0001	2.60	2.41	2.79	<0.0001	2.34	2.22	2.48	<0.0001
Depression	1.34	1.31	1.37	<0.0001	2.05	1.85	2.26	<0.0001	1.99	1.85	2.14	<0.0001
Anxiety	1.36	1.33	1.39	<0.0001	1.82	1.61	2.05	<0.0001	2.15	1.98	2.34	<0.0001
Diabetes	1.37	1.35	1.39	<0.0001	1.93	1.79	2.08	<0.0001	1.77	1.67	1.88	<0.0001
Hyperlipidemia	1.21	1.20	1.23	<0.0001	1.09	1.02	1.16	0.0122	1.16	1.10	1.22	<0.0001
Hypertension	1.31	1.29	1.33	<0.0001	1.71	1.60	1.83	<0.0001	1.63	1.56	1.72	<0.0001
Obesity	1.38	1.35	1.42	<0.0001	2.17	1.93	2.45	<0.0001	1.89	1.73	2.08	<0.0001
Cerebrovascular disease	1.41	1.37	1.46	<0.0001	2.08	1.84	2.35	<0.0001	2.01	1.81	2.23	<0.0001
Peripheral vascular disease	1.47	1.43	1.52	<0.0001	2.38	2.10	2.69	<0.0001	1.95	1.75	2.16	<0.0001
Any of the above comorbidities^a^	1.49	1.47	1.51	<0.0001	2.03	1.87	2.19	<0.0001	1.84	1.75	1.94	<0.0001

Note: For each comorbidity, the reference group is the patients who do not have the respective comorbidity

*IRR* incidence rate ratio, *CL* confidence limit

^a^Any of the above comorbidities means if patients have any of the either comorbidities mentioned above

Data for outpatient visits, hospitalization visits and emergency room visits for each of the comorbidities for the controlled variables are not reported here, but these can be made available upon request

### Direct costs

Table 3 presents the adjusted direct costs for patients with and without comorbidities, and incremental adjusted direct costs. In general, psoriasis patients with comorbidities had higher adjusted direct costs compared with psoriasis patients without comorbidities. Among the group of psoriasis patients with comorbidities, the annual adjusted direct costs for each comorbidity was high with higher estimates for those with PsA and peripheral vascular disease ($25,035.9 and $24,871.7, respectively). The highest mean incremental adjusted direct costs between the two groups of patients were observed in PsA ($9914.3), peripheral vascular diseases ($8386.5) and cardiovascular disease ($8275.1) (Table 3).

**Table 3:** Adjusted annual direct costs ($) by type of comorbidity

Comorbidity	No. of patients with comorbidity	Adjusted costs for patients with comorbidity	No. of patients without comorbidity	Adjusted costs for patients without comorbidity	Incremental adjusted costs	Lower 95% CL	Upper 95% CL
Psoriatic Arthritis	5557	25,035.9	50,849	13,376.2	9914.3	9215.8	10,612.7
Cardiovascular disease	9992	22,287.1	46,414	12,853.8	8275.1	5900.6	7630.0
Depression	4388	20,270.7	52,018	14,040.2	6765.3	1188.2	2081.8
Anxiety	3148	17,569.3	53,258	14,344.9	4181.2	6682.3	10,090.7
Diabetes	8031	22,052.1	48,375	13,275.2	6911.4	6271.3	7551.5
Hyperlipidemia	18,911	16,502.7	37,495	13,527.3	1635.0	6254.9	7024.3
Hypertension	19,365	18,465.9	37,041	12,464.5	4522.3	5754.1	8255.9
Obesity	2755	20,827.1	53,651	14,201.2	6564.5	5419	7710.1
Cerebrovascular disease	2122	23,410.0	54,284	14,177.5	7005.0	2950.6	5411.9
Peripheral vascular disease	2032	24,871.7	54,374	14,138.2	8386.5	7461.2	9089
Any of the comorbidities^a^	35,644	17,425.8	20,762	9544.5	6639.6	4033.5	5011.2

Note: The adjusted costs were predicted based on the model results using the recycled prediction method [38] for patients with and without comorbidities using the study sample. The incremental adjusted costs are differences in the adjusted costs between patients with and without comorbidities. The 95% CLs were calculated using bootstrapping method for both direct and indirect costs

^a^Any of the above comorbidities means if patients have any of the either comorbidities mentioned above

### Indirect costs associated with short-term disabilities

Among the group of psoriasis patients with comorbidities, the annual adjusted indirect costs for each comorbidity was particularly high for those with cerebrovascular disease and obesity ($2501.5 and $2293.4, respectively). Psoriasis associated with some of the comorbidities had higher adjusted indirect costs of short-term disabilities than those for patients without comorbidities. The mean incremental adjusted indirect costs for psoriasis patients with cerebrovascular disease, obesity, peripheral vascular disease, depression, and PsA were $1333, $1195, $9,94.9, $996.6, and $403.5, respectively (Table 4).

**Table 4:** Adjusted annual indirect costs ($) due to short-term disabilities by type of comorbidity

Comorbidity	No. of patients with comorbidity	Adjusted costs for patients with comorbidity	No. of patients without comorbidity	Adjusted costs for patients without comorbidity	Incremental adjusted costs	Lower 95% CL	Upper 95% CL
Psoriatic arthritis	516	1346.7	5362	860.0	403.5	−44.9	852.0
Cardiovascular disease	543	1635.0	5335	828.2	817.8	314.1	1321.6
Depression	355	2112.7	5523	825.0	996.6	431.6	1561.6
Anxiety	267	1344.2	5611	881.8	267.8	−300.9	832.8
Diabetes	570	1816.3	5308	804.7	928.0	400.1	1456.0
Hyperlipidemia	1624	1163.2	4254	803.3	356.5	70.0	643.0
Hypertension	1373	1319.0	4505	775.9	576.5	233.7	919.3
Obesity	189	2293.4	5689	856.6	1195.1	16.1	2374.1
Cerebrovascular disease	81	2501.5	5797	880.4	1333.1	−181.1	2847.2
Peripheral vascular disease	64	1912.2	5814	891.6	994.9	−789.2	2779.0
Any of the above^a^	3123	1191.9	2755	575.0	614.1	390.0	838.2

Note: The adjusted costs were predicted based on the model results using the recycled prediction method [38] for patients with and without comorbidities using the study sample. The incremental adjusted costs are differences in the adjusted costs between patients with and without comorbidities. The 95% CLs were calculated using bootstrapping method for both direct and indirect costs

^a^Any of the above comorbidities means if patients have any of the either comorbidities mentioned above

## Discussion

This study, using a large real-world commercial database in the US, informs on the longitudinal and annual incremental direct and indirect costs of comorbidities in psoriasis. Our results indicated that hypertension, hyperlipidemia, cardiovascular disease, diabetes, and PsA are common comorbidities in psoriasis patients. Patients having moderate to severe psoriasis had higher comorbidity burden compared to those having mild disease. Moreover, presence of comorbidities among these patients is associated with an incremental economic burden compared to psoriasis patients without comorbidities.

Studies reporting incremental economic burden of comorbidities in psoriasis patients compared to those without comorbidities are very limited in the literature. Kimball et al. estimated the incremental economic burden associated with comorbidities in patients with psoriasis using data from the Ingenix Impact National Managed Care Database (IMPACT) (1999–2004) [22]. Resource utilization and costs during the 6-month follow-up period were compared for patients with comorbidity vs those without. In this study, it was reported that hyperlipidemia (27.3%) and hypertension (25.4%) were the most common comorbidities followed by depression (9.2%), diabetes (8.7%) and cardiovascular disease (8.6%). Our study also found that hyperlipidemia (33.53%) and hypertension (34.33%) followed by cardiovascular diseases (17.71%), diabetes (14.24%) and depression (7.78%) were the most frequently reported comorbidities in psoriasis patients. Furthermore, Kimball et al. reported that patients with comorbidities were more likely to experience urgent care, greater hospitalization rates, and outpatient visits than patients without comorbidities. Similarly, our study also reported higher healthcare resource utilization burden for psoriasis patients with comorbidities vs. those without comorbidities.

Kimball et al. also reported that conditions like cerebrovascular disease, peripheral vascular disease, cardiovascular disease, and PsA are associated with the highest adjusted incremental costs in psoriasis patients among investigated comorbidities [22]. Our results corroborate these findings as PsA, cardiovascular disease, and peripheral vascular disease had the highest incremental adjusted direct costs in our study. Our study also reported substantial incremental economic burden of psychological comorbidities (depression and anxiety) among psoriasis patients, which was not previously reported in the literature. Kimbal et al. in their analysis used old data till 2004, when biologics were not available for PsO. So our study can be considered as an update after introduction of biologics to treat PsO.

Feldman et al. reported that compared with matched psoriasis and PsA free controls, moderate-to-severe psoriasis patients with comorbid PsA had higher comorbidity and health care utilization and costs [12]. Crown et al. reported that psoriasis patients with selected comorbidities have significantly higher mean total healthcare expenditures compared to non- psoriasis persons with the same comorbidities [8]. Although there are differences between our study and those available in the literature (e.g., in terms databases used, study timelines, patient populations etc.), major findings are on similar lines.

It is known that indirect costs are associated with a high economic burden in psoriasis. Some recent U.S. studies have elucidated several components of burden of disease in psoriasis patients and a discernible proportion of economic burden is constituted by indirect costs [4, 6]. Similar reports have also been published from Germany, where average annual costs for PsA patients increased from €3150 per patient, to approximately €5500–11,075 per patient, when indirect costs are also considered [18]. Authors have also reported that indirect costs in psoriasis is a major problem in everyday life [35] and the annual indirect costs may exceed the direct costs [33]. Our study reported the incremental indirect costs due to short-term disabilities in psoriasis patients with comorbidities which have not been investigated before. Hence this finding from our study is an important addition to the existing literature.

The impact of comorbidities on health care cost may be attributable to the additional medical resources consumed for treating these illnesses. In addition, the coexistence of psoriasis and another illness may exacerbate the deleterious effects of each condition. The presence of comorbidities in patients with psoriasis may complicate the management of both diseases. Our findings can provide a more complete picture to health care providers and policy makers of the economic implications of psoriasis. These findings suggest that comorbidities deserve serious consideration, both clinically and economically, in the overall treatment and management plans for patients with psoriasis.

### Limitations

Our study is subject to several limitations. When the study was designed, our investigation was the first claims database analysis aimed at estimating the direct costs of comorbidities in psoriasis, post the introduction of biologics. However, the analysis was not adjusted by type of psoriasis treatment. The effect of treatment on the costs of comorbidities should be investigated in the future with recent datasets and patients treated with new and highly effective systemic treatments of psoriasis. Due to the observational design, the analysis may have been affected by unobserved differences between patients with comorbidities versus those without. Any coding inaccuracies may lead to misclassification, resulting in overestimation or underestimation of the number of psoriasis cases with comorbidities in the database. Psoriasis patients were categorized based on their drug therapy rather than the clinical measures of disease severity (such as Psoriasis Area and Severity Index). Drug treatment as a proxy of disease severity is commonly used in commercial claims database analyses and appears to be an acceptable method of classifying patients into disease severity categories [36]. Moreover, owing to the retrospective design of the study and missing confounding, any causal relation of comorbidities and psoriasis could not be made. The data used in this analysis was limited to patients with commercial and Medicare supplemental insurances; therefore, the findings may not be generalizable to psoriasis patient population and the population outside the US. The results were controlled with respect to age, gender, and psoriasis severity using treatment type as proxy, but other possible covariates (income, co-payment, treatment initiation, treatment type, baseline expenditures, CCI score) could also have affected the results. This research is restricted only to the economic burden associated with the presence of comorbidities and does not consider other key aspects of the burden such as humanistic or caregiver burden of the disease. Indirect costs included the value of lost work time due to short-term disabilities only and were estimated for a subset of patients for whom data were available. This warrants further investigation of the economic impact of comorbid psoriasis in terms of work productivity with consideration to absenteeism from work and long-term disabilities due to the disease.

## Conclusion

Psoriasis patients with comorbidities used more healthcare resources, and had higher direct costs and indirect costs due to short- term disability compared with those without comorbidities. These findings underline the need for adequate clinical and economic considerations when treatment algorithms are devised for psoriasis patients with comorbidities. Incorporating a step for screening psoriasis patients for comorbidities may pave the way for better patient outcomes and reduced burden of disease, however blanket screening recommendations would be premature without careful analysis of the effectiveness, benefits and costs of such screening.

### Recommendations for future research

The present study highlights economic burden due to presence of comorbidities in patients with psoriasis. Future research could target to assess additional indirect and intangible costs of comorbidities in psoriasis as well as effect of newly launched systemic treatments in alleviating this burden.

## Abbreviations

CCI: Charlson Comorbidity Index
HMO: Health Maintenance Organization
HPM: Health and Productivity Management
ICD: International Classification of Diseases
IRR: Incidence rate ratio
